# Altered Network Timing in the CA3-CA1 Circuit of Hippocampal Slices from Aged Mice

**DOI:** 10.1371/journal.pone.0061364

**Published:** 2013-04-08

**Authors:** Daniel J. Kanak, Gregory M. Rose, Hitten P. Zaveri, Peter R. Patrylo

**Affiliations:** 1 Department of Physiology, Southern Illinois University School of Medicine, Carbondale, Illinois, United States of America; 2 Department of Anatomy, Southern Illinois University School of Medicine, Carbondale, Illinois, United States of America; 3 Center for Integrated Research in Cognitive & Neural Sciences, Southern Illinois University, Carbondale, Illinois, United States of America; 4 Department of Neurology, Yale University, New Haven, Connecticut, United States of America; Consejo Superior de Investigaciones Cientificas - Instituto Cajal, Spain

## Abstract

Network patterns are believed to provide unique temporal contexts for coordinating neuronal activity within and across different regions of the brain. Some of the characteristics of network patterns modeled *in vitro* are altered in the CA3 or CA1 subregions of hippocampal slices from aged mice. CA3–CA1 network interactions have not been examined previously. We used slices from aged and adult mice to model spontaneous sharp wave ripples and carbachol-induced gamma oscillations, and compared measures of CA3–CA1 network timing between age groups. Coherent sharp wave ripples and gamma oscillations were evident in the CA3–CA1 circuit in both age groups, but the relative timing of activity in CA1 stratum pyramidale was delayed in the aged. In another sample of aged slices, evoked Schaffer collateral responses were attenuated in CA3 (antidromic spike amplitude) and CA1 (orthodromic field EPSP slope). However, the amplitude and timing of spontaneous sharp waves recorded in CA1 stratum radiatum were similar to adults. In both age groups unit activity recorded juxtacellularly from unidentified neurons in CA1 stratum pyramidale and stratum oriens was temporally modulated by CA3 ripples. However, aged neurons exhibited reduced spike probability during the early cycles of the CA3 ripple oscillation. These findings suggest that aging disrupts the coordination of patterned activity in the CA3–CA1 circuit.

## Introduction

Many aspects of cognition, including memory, are thought to involve dynamic interactions among ensembles of neurons that are coordinated by different types of patterned network activity 1]. Area CA3 is an important source of some of the network patterns generated within the hippocampus. For example, cholinergic disinhibition of recurrent collaterals 2] leads to irregular population bursts in CA3. These bursts generate negative deflections in CA1 stratum radiatum called “sharp waves”, which are associated with high frequency (∼200 Hz) “ripple” oscillations in stratum pyramidale 3,4]. The temporally-compressed reactivation of place-related firing patterns during sharp wave ripples 5,6], and the propagation of this information through the hippocampal formation 7], are believed to be important steps in memory consolidation [8], [9], [10], [11]. Gamma oscillations emerging in the CA3 subregion can also entrain network activity in CA1 12,13], and this mode of coupling is thought to assist memory retrieval during active exploration 14,15]. Therefore, by coordinating neuronal activity between subregions, CA3-generated network patterns might facilitate information transfer to CA1.

The *in vitro* hippocampal slice preparation is commonly used as a model system to study sharp wave ripples [16], [17], [18] and gamma oscillations 19]. Earlier studies have reported age-related reductions in the intensity of ripple oscillations [20] or gamma oscillations [21], [22], [23] in the field potentials recorded from CA3 or CA1, but none have examined the temporal relationship of network patterns between subregions. Given the reported changes in Schaffer collateral synaptic excitation [24], [25], [26] and GABAergic interneurons [27], [28], [29] in the CA1 subregion of aged rodents, we hypothesized that aging might disrupt the capacity of CA3-generated network patterns to entrain activity in CA1. To test our hypothesis, we examined spontaneous sharp wave ripples and carbachol-induced gamma oscillations modeled in hippocampal slices from aged and adult mice, with a particular focus on measures of CA3–CA1 network timing.

## Methods

### Ethics Statement

This study was done in accordance with the recommendations of the Guide for the Care and Use of Laboratory Animals of the National Institutes of Health. The protocol was approved by the Southern Illinois Institutional Animal Care and Use Committee (permit number: 10–013). Mice were deeply anesthetized with sodium pentobarbital anesthesia (50 mg/kg, i.p.) prior to sacrifice.

### Animals

Experiments were performed in male 129/C57BL6 mice, 4.0±0.1 months old (“adult”, n = 22) and 21.7±0.3 months old (“aged”, n = 18). The mice had *ad libitum* access to food and water, and were kept on a 12 hour/12 hour light/dark cycle (lights on at 0800). Experiments were performed during the animals' light cycle.

### Slice preparation

Mice anesthetized with sodium pentobarbital (50 mg/kg, i.p.) were transcardially perfused (∼20 mL, 40 mL/min) with cold (1–2 °C) sucrose-based artificial cerebo-spinal fluid (sucrose-aCSF; composition in mM: 85 NaCl, 75 sucrose, 26 NaHCO_3_, 25 dextrose, 3 KCl, 2.5 CaCl_2_, 1.4 NaH_2_PO_4_, 1.3 MgSO_4_) saturated with carbogen (95/5 O_2_/CO_2_). Horizontally-cut slices (400 µM) were prepared from the ventral one-half of the hippocampus and then transferred to an interface-style recording chamber (32–34 °C) perfused (3.5 mL/min) with a 1∶1 solution of sucrose-aCSF and normal-aCSF (composition in mM: 124 NaCl, 26 NaHCO_3_, 10 dextrose, 3 KCl, 2.5 CaCl_2_, 1.4 NaH_2_PO_4_, 1.3 MgSO_4_) for 30 minutes, followed by normal-aCSF. Experiments began at least 60 minutes thereafter.

### Electrophysiology

Field potentials were recorded using silver/silver-chloride electrodes encapsulated in 2–4 MΩ borosilicate glass micropipettes (P-97, Sutter Instrument Company, Novato, CA) filled with normal-aCSF. Signals were amplified (100×) and bandpass-filtered (0.1 Hz–3 kHz) using differential amplifiers (DAM50, World Precision Instruments, Sarasota, FL; DP-304, Warner Instruments, Hamden, CT). Unit recordings were amplified (DC-coupled, 100×) using a high impedance bridge-balance amplifier (AxoClamp 2B, Axon Instruments, Foster City, CA). Juxtacellular recordings were obtained “blindly” from neurons in CA1 stratum pyramidale and stratum oriens. Extracellular units were initially detected by monitoring an audio signal for brief “ticks” occurring during spontaneous sharp waves. The pipette was advanced further to obtain a stable, 100–200 MΩ resistance recording, which was inferred from the voltage step responses elicited by 500 ms, 0.1 nA current pulses. This was taken to indicate contact or close proximity with the cell membrane. Signals were digitized at 10101 Hz or 5000 Hz with PCI-6221 analog-to-digital converters (National Instruments, Austin, TX) and logged to a computer hard drive using custom MATLAB scripts (R2007a, The MathWorks, Natick, MA).

### Offline sharp wave detection

Sharp waves were segmented from the continuous recordings using the following procedure. First, CA3 field potentials were digitally filtered (50 Hz lowpass, 172-pole FIR equiripple design) using the phase-conserving *filfilt* command in MATLAB. Hilbert transforms were then applied to identify amplitude threshold-crossings (2.5 times the signal's root mean square power; RMS) that coincided with positive-going zero-crossings in the phase-shifted component of the Hilbert transform. The phase criterion established a temporal reference for aligning CA3 sharp waves. These indices were then used to segment CA3 and CA1 sharp waves from the raw recordings, and the results were confirmed by visual inspection.

### Coherence

For the time series 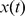 and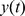, coherence 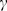 is defined as



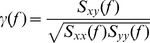
where 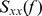 and 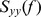 are the auto-power spectral densities and 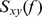 is the complex-valued cross-power spectrum. Magnitude squared coherence (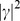, MSC) provides a frequency-dependent measure of the strength of the linear association between two time-series. MSC is similar to Pearson's Product Moment Correlation Coefficient or r-value, though with a frequency index and a range of 0 to 1. For CA3 and CA1 field potential recordings we estimated coherence as 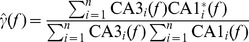
where 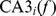 and 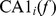 are sample spectra calculated by applying 2^14^ point fast Fourier transforms to 60 one-second non-overlapping Hanning-weighted data segments, and 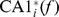 denotes the complex conjugate of 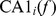. To establish a significance threshold we performed 1000 iterations on randomly generated Gaussian white noise and measured the one-tailed 95% upper confidence limit on the distribution of peak MSC values (0.12). Error thresholds using randomly shuffled CA3 and CA1 data segments yielded similar values (mean aged = 0.12±0.0038, mean adult = 0.12±0.0024). For statistically significant values of MSC, we calculated the phase difference 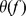 between CA3 and CA1 as 
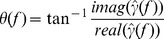
where 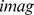 and 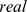 are the imaginary and real components of the complex coherence estimate. Discontinuities in the phase spectra were corrected using the command *unwrap* in MATLAB.

### Unit timing

CA3 sharp wave ripples and CA1 unit activity were segmented from 180 s segments of spontaneous recording using the procedure described in the previous section. Ripple oscillations were isolated using a digital bandpass filter (100–500 Hz, 82-pole FIR with Hamming window). Units were detected as amplitude threshold-crossings exceeding 6 times the RMS power of an event-free epoch of data. Unit time delays were measured relative to the negative peaks of CA3 ripple oscillations. Spike probability histograms were constructed by counting the number of units in 1 ms delay bins and dividing by the total number of detected ripples. The histograms were normalized by dividing by the peak probabilities. We also calculated the phase difference between units and individual ripple cycles from Hilbert transforms. Unfortunately, we used a relatively long sampling period (0.2 ms) to digitize unit activity. While this is more than adequate for the analysis of unit timing given the ∼4–5 ms period of the ripple cycle and the 1 ms bin width of the spike probability histograms, we were unable to differentiate pyramidal cells from interneurons based on waveform characteristics.

### Statistical Analysis

Statistical testing was performed in SPSS (version 16, BM, Armonk, NY) and in MATLAB using the CircStat toolbox 30]. We used independent samples t-tests to compare network characteristics between age groups. Corrected t-tests were used in cases where Levene's tests indicated there was a significant difference in error variance between age groups. Non-parametric Mann-Whitney U-tests were used in cases where Kolmogorov-Smirnov tests indicated that the samples were not normally distributed. The Mann-Whitney U-test was also used to compare unit firing delays between age groups since the probability distributions were not normally distributed. To compare phase differences between age groups we used the Watson-Williams test for circular data. Age-related differences in antidromic and orthodromic stimulus response series were assessed using two (*age*) x nine (*stimulus intensity*) repeated measures ANOVAs – Greenhouse-Geisser corrections were applied since Mauchly's tests indicated there was significant violation of the sphericity assumption. The type-1 error rate was fixed at 0.05 for all statistical tests. Values shown in the text are mean ± SEM.

## Results

**Figure 1:**
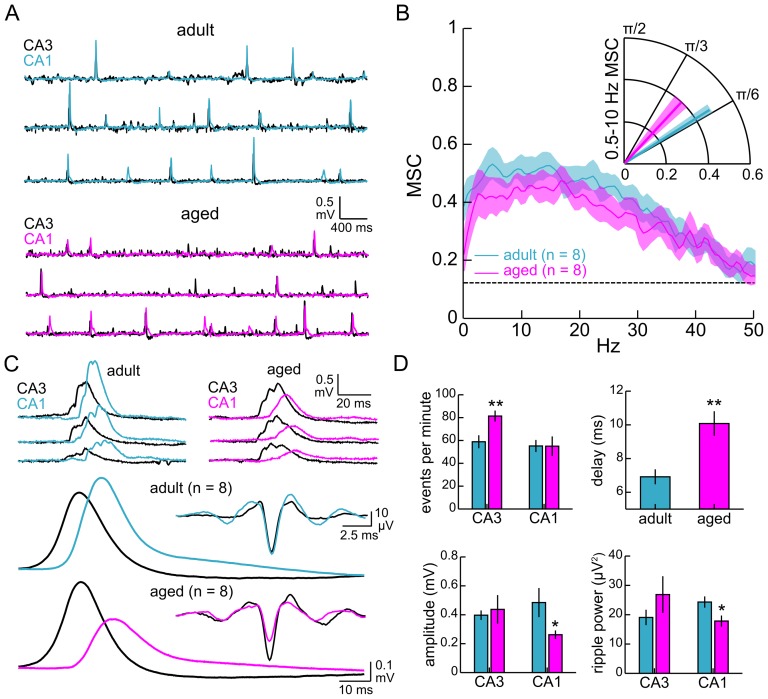
Aging alters the timing and magnitude of sharp wave ripple activity in CA1. **A**. Spontaneous sharp wave ripples recorded from stratum pyramidale of CA3c (*black*) and mid-distal CA1 (*blue-adult; magenta-aged*). Representative traces from three mice per age group are shown. **B**. Magnitude squared coherence (MSC) was not significantly different between age groups, although the phase coherence between CA3 and CA1 was increased in aged slices compared to adult slices (*inset*). Dashed line represents the 95% significance threshold (0.12). Error bands are SEM or circular SEM. The inset shows phase coherence in the 0.5–10 Hz frequency band, with mean MSC as the vector lengths and circular mean phase coherence as the vector angles. **C**. Top: Examples of sharp wave ripples segmented from the continuous recordings. The traces were selected at random from the adult and aged data sets and then sorted by amplitude for the figure. Bottom: Waveform averages of sharp waves and ripple oscillations. Ripples were filtered (100–500 Hz) and temporally aligned by their peak negative voltages before averaging. **D**. Summary comparison of sharp wave and ripple characteristics between aged and adult slices. Top left: frequency (events per minute). Top right: CA3–CA1 delay (peak to peak time difference). Bottom left: waveform amplitude. Bottom right: ripple RMS power. Sharp waves were more frequenct in aged CA3, whereas CA1 sharp wave ripple activity was temporally delayed and smaller in magnitude in aged slices compared to adult slices. *p<0.05; **p<0.01.

Consistent with other *in vitro* studies [16], [17], [18], spontaneously occurring sharp waves were observed in slices prepared from the ventral aspect of the hippocampus (aged = 10 slices from 8 mice; adult = 12 slices from 8 mice). The sharp waves were not hyperexcitable events related to epileptiform bursts since they were abolished in the presence of the GABA_A_ receptor antagonist bicuculline (1–2 µM, n = 5 slices, *data not shown*). In stratum pyramidale (closer to stratum oriens), sharp waves appeared as positive deflections in the field recordings (Fig. 1A and 1C), with CA1 sharp waves delayed by 5–25 ms relative to sharp waves in CA3. Knife cuts through proximal CA1 abolished or dramatically reduced sharp waves in CA1 without affecting activity in CA3 (n = 3, *data not shown*), demonstrating the importance of the Schaffer collaterals in entraining CA1 sharp waves.

To measure the strength of the association between CA3 and CA1 sharp wave activity we estimated magnitude squared coherence (MSC), which is interpreted as a frequency-dependent r-value. Moderate, statistically significant values of MSC(≥0.12) were broadly distributed from DC to approximately 50 Hz (Fig. 1B). Since peak power occurred at frequencies below 10 Hz (aged = 6.3±1.0 Hz, adult = 5.3±0.8 Hz, t_14_ = 1.10, p = 0.29), we focused on activity in the 0.5–10 Hz frequency band. Sharp wave MSC was not significantly different between age groups (aged = 0.43±0.046, adult = 0.53±0.051, t_14_ = 1.57, p = 0.14). However, we found an increase in the phase difference between CA3 and CA1 sharp wave activity in aged slices compared to adult slices (aged = 0.82±0.087 radians, adult = 0.56±0.043 radians, t_14_ = 2.52, p = 0.024; Fig. 1B, *inset*). Since peak frequencies in the coherence spectra were ambiguous, we derived time delays for the phase differences using the peak power frequencies noted above, and obtained values of 20.7 ms in aged slices and 16.8 ms in adult slices. That is, on average, CA1 sharp wave activity was delayed in the aged by 3.9 ms when compared to adults.

Some of the activity contributing to the 0.5–10 Hz frequency band might not be related to sharp wave activity. Therefore, we segmented individual sharp waves from the continuous recordings to measure timing more directly (Fig. 1C, *upper traces*). The number of sharp waves detected in 60 s epochs of recording from CA3 was increased in the aged slices (aged = 81±5, adult = 59±6, t_14_ = 3.02, p = 0.0092), but CA1 sharp wave numbers were comparable between age groups (aged = 55±9, adult = 55±5, t_14_ = 0.019, p = 0.99; Fig 1D, *upper left*). Consistent with the phase coherence analysis, the peak-to-peak time difference between CA3 and CA1 sharp waves was greater in the aged (aged = 10.1±0.7 ms, adult = 6.9±0.5 ms, t_14_ = 3.48, p = 0.0037Fig 1D, *upper right*), with an average difference of 3.2 milliseconds. Since there was a large discrepancy between these values and the delays estimated from the phase coherence analysis, we also calculated time delays from the peaks in the cross-correlations between CA3 and CA1, and obtained values similar to those derived from the direct measure (aged = 10.4±1.1 ms, adult = 6.8±0.5 ms, t_14_ = 2.99, p = 0.0097; Fig. S1).

In addition to the timing alteration, the peak amplitude of CA1 sharp waves was smaller in aged slices (aged = 0.26±0.030 mV, adult = 0.48±0.10 mV, U = 12, p = 0.036), whereas CA3 sharp wave amplitudes were similar between age groups (aged = 0.44±0.099 mV, adult = 0.40±0.033 mV, t_14_ = 0.71, p = 0.38; Fig. 1D, *lower left*). Ripple oscillations in CA1 are also reportedly smaller in slices from aged mice [20]. To compare ripple magnitude between age groups we used a digital filter (100–500 Hz, 82-pole FIR with Hamming window) to isolate ripples from the segmented sharp waves (Fig. 1C, *lower right*), and measured RMS power for the portion of the signal that exceeded a threshold (6× RMS of a 50 ms epoch of event free data). Consistent with the findings reported by Hermann and colleagues [20], CA1 ripples were also smaller in the aged slices examined in our study (aged = 17.84+1.89 µV^2^, adult = 24.32+1.92 µV^2^, t_14_ = 2.41, p = 0.031). No change in CA3 ripple power was observed (aged = 21.44±3.35 µV^2^, adult = 19.06±2.64 µV^2^, t_14_ = 1.15, p = 0.27, Fig. 1D, *lower right*). Additionally, there were no group differences in the frequencies estimated from the autocorrelation functions of CA3 ripples (aged = 200.1±1.0 Hz, adult = 201.2±1.2 Hz, t_14_ = 0.73, p = 0.48) or CA1 ripples (aged = 204.1±4.4 Hz, adult = 211.12±2.6 Hz, t_14_ = 1.36, p = 0.20, *data not shown*).

**Figure 2:**
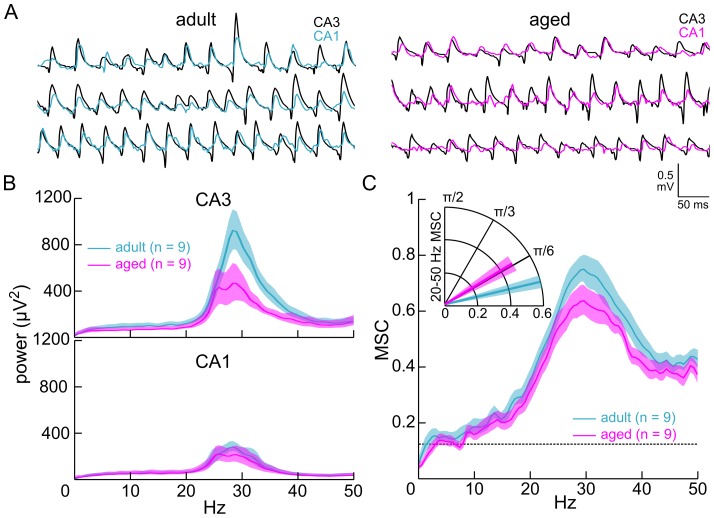
Age-related changes in carbachol-induced gamma oscillations. **A**. Coherent gamma oscillations recorded from stratum pyramidale of CA3c (*black*) and mid-distal CA1 (*blue-adult; magenta-aged*) of hippocampal slices exposed to bath-applied carbachol (10 µM) for 45–90 minutes. The traces were recorded from three different mice per age group. **B**. Power spectral density in CA3 (*top*) and CA1 (*bottom*). Power was significantly reduced in area CA3, but not area CA1, of aged slices. **C**. Magnitude squared coherence (MSC) estimates revealed significantly reduced gamma coherence as well as an increased CA3–CA1 phase difference in slices from aged mice. The Inset shows phase coherence in the 20–50 Hz frequency band, with mean MSC as the vector lengths and circular mean phase coherence as the vector angles. Dashed line represents the 95% significance threshold (r = 0.12). Error bands are SEM or circular SEM.

Next, we examined CA3–CA1 network timing in slices exposed to the cholinergic agonist carbachol. Within approximately 5 minutes of continuous bath exposure to 10 µM carbachol, sharp wave ripples were abolished in the field potentials recorded from stratum pyramidale, as previously shown 16], and coherent gamma oscillations gradually emerged, consistent with earlier reports 12,13,15] (Fig. 2A). After allowing 45–90 minutes for gamma power to stabilize, five minute recordings of field potential activity were obtained from slices prepared from both ventral and middle hippocampus (aged = 24 slices from 9 mice; adult = 30 slices from 9 mice). Offline measures of gamma power confirmed that the oscillations were stable over the duration of these recordings (CA3 power coefficient of variation: aged = 0.075, adult = 0.069, t_16_ = 0.31, p = 0.42; CA1 power coefficient of variation: aged = 0.077, adult = 0.070, t_16_ = 1.42, p = 0.18). As reported previously [22], gamma power was reduced in aged CA3 (aged = 239.74+46.78 µV^2^, adult = 326.73+39.18 µV^2^, t_16_ = 2.16, p = 0.046, Fig. 2B, *top*). However, CA1 gamma power was not statistically different between age groups in our study (aged = 82.25±18.89 µV^2^, adult = 102.45±10.90 µV^2^, t_16_ = 1.67, p = 0.12; Fig. 2B, *bottom*). In contrast to sharp wave coherence, CA3–CA1 gamma coherence was significantly reduced in the aged (aged = 0.48±0.034, adult = 0.60±0.021, t_16_ = 2.48, p = 0.023, Fig. 2C). This was accompanied by a larger phase difference when compared to adults (aged = 0.54±0.098 radians, adult = 0.23±0.059 radians, t_16_ = 2.79, p = 0.013, Fig. 2C, *inset*). With peak frequencies of 31.7 Hz in the aged and 30.8 Hz in the adults (t_16_ = 0.61, p = 0.55), the phase differences correspond to temporal delays of 2.7 ms and 1.2 ms, a difference of 1.5 ms. Similar values were observed in the time delays measured from the peaks in the cross-correlations between CA3 gamma and CA1 gamma (aged = 2.9±0.3 ms, adult = 1.6±0.3 ms, t_16_ = 2.90, p = 0.010, Fig. S2). Therefore, gamma activity also appears to be delayed in aged CA1 stratum pyramidale.

**Figure 3:**
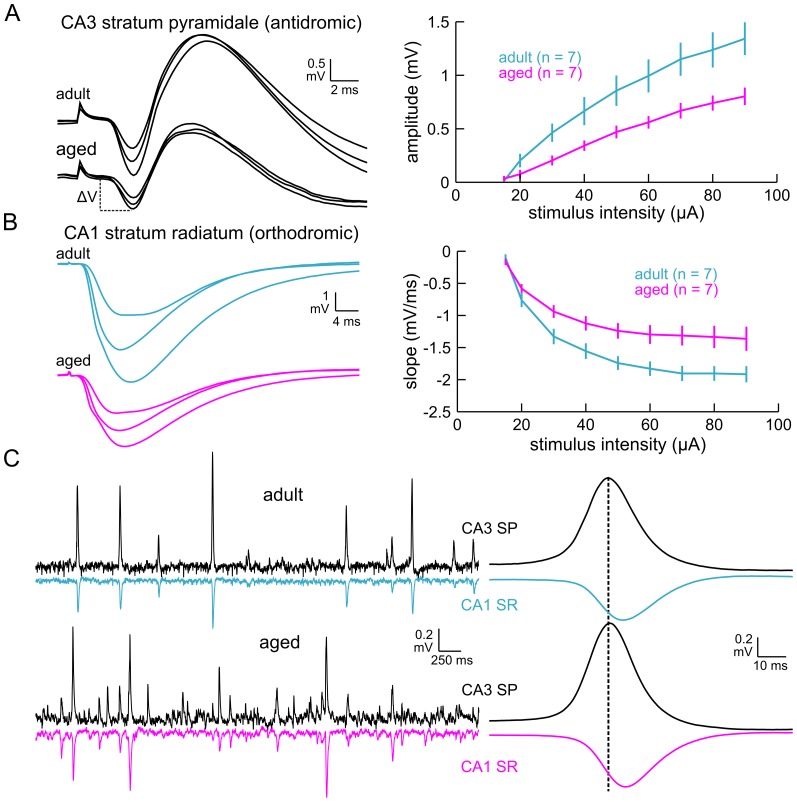
Age-related alterations evident in Schaffer collateral evoked responses are not manifested in the spontaneous sharp wave activity recorded in CA1 stratum radiatum. A. Left: Putative antidromic population spikes recorded in CA3c stratum pyramidale in an adult slice (*top traces*) and an aged slice (*bottom traces*). The traces are averaged responses evoked by electrical stimulation in proximal CA1 stratum radiatum using 30, 60, and 90 µA stimulus intensities (n = 3 each). Right: Antidromic spike amplitude was significantly attenuated in aged slices compared to adult slices. B. Left: Orthodromic field EPSPs recorded in mid-distal CA1 stratum radiatum. The traces are averaged responses evoked by electrical stimulation in proximal CA1 stratum radiatum using 30, 60, and 90 µA stimulus intensities (n = 3 each). Right: field EPSP slope (10–50% of peak amplitude) was significantly reduced in the aged. C. Left: Spontaneous sharp waves recorded from CA3 stratum pyramidale and CA1 stratum radiatum in a representative slice from each age group. Sharp waves appear as negative deflections in the stratum radiatum recordings. Right: Waveform averages of sharp waves segmented from the continuous recordings. The timing and magnitude of sharp waves in CA1 stratum radiatum were comparable between age groups. Error bars in panels A and B are SEM. SP-stratum pyramidale. SR-stratum radiatum.

The age-related alterations in network timing might reflect Schaffer collateral dysfunction. Therefore, in another group of slices (aged = 17 slices from 7 mice, adult = 16 slices from 7 mice), we delivered electrical stimulation (0.1 ms pulse width, 0.05 Hz, 15–90 µA) in stratum radiatum of proximal CA1 to activate Schaffer collateral afferents and measured 1) the latency and amplitude of the presumptive antidromic population spike recorded in stratum pyramidale of CA3c and 2) the initial slope (10–50% of peak amplitude) of the orthodromic field EPSP recorded simultaneously in stratum radiatum of mid-distal CA1. Spike latencies in CA3 were consistent over the range of stimulus intensities applied (consistent with an antidromic response) and were not different between age groups (aged = 3.8±0.2 ms, adult = 3.6±0.1 ms, t_12_ = 0.90, p = 0.38). However, antidromic spike amplitudes in CA3 were significantly smaller in the aged (*age* × *stimulus intensity* interaction effect, F_1.38, 16.54_ = 7.39, p = 0.0095, repeated measures ANOVA with Greenhouse-Geisser correction; Fig. 3A). Additionally, field EPSP slopes recorded in stratum radiatum of CA1 were significantly reduced in aged slices compared to adult slices (*age* × *stimulus intensity* interaction effect, F_1.91, 22.96_ = 3.93, p = 0.036, repeated measures ANOVA with Greenhouse-Geisser correction; Fig. 3B). However, despite the differences in electrically-evoked measures of Schaffer function, aging did not alter the timing or amplitude of spontaneous sharp waves recorded in CA1 stratum radiatum (CA3–CA1 peak to peak time delay: aged = 5.6±0.5 ms, adult = 5.6±0.7 ms, t_12_ = 0.027, p = 0.98; amplitude: aged = 0.27±0.032 mV, adult = 0.22±0.012 mV, t_12_ = 1.46, p = 0.17, Fig 3C). These results suggest that while electrical stimulation can unmask Schaffer collateral impairments, synaptic excitation appears be sufficiently intact to generate spontaneous sharp wave activity in stratum radiatum of aged CA1.

**Figure 4:**
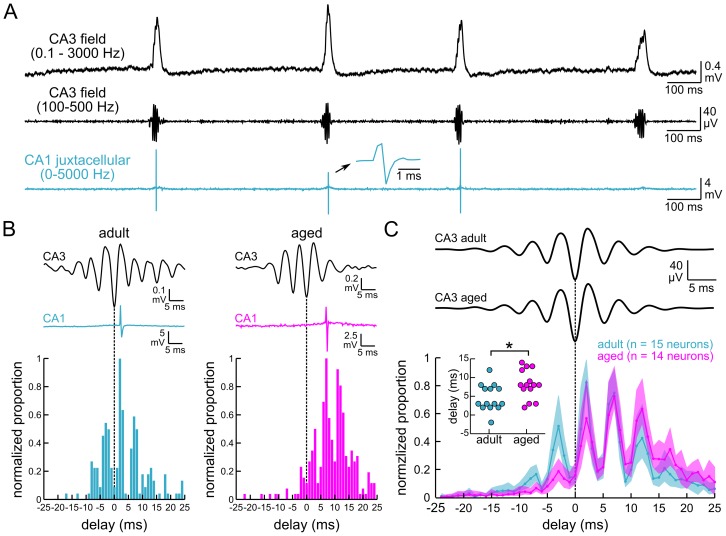
Ripple-associated unit firing in CA1 is delayed in slices from aged mice. **A**. Top: Spontaneous sharp wave ripples recorded from CA3 stratum pyramidale. Middle: Ripple oscillations isolated from the wideband recording using a bandpass filter (100–500 Hz). Bottom: Juxtacellular unit activity recorded from a CA1 neuron near the stratum pyramidale/stratum oriens border. The unit shown on the expanded time scale is an example of a truncated waveform that resulted from low sampling resolution (5 kHz). **B**. Top: CA3 ripple oscillations recorded from an adult slice (*left*) and an aged slice (*right*). The corresponding CA1 unit activity is shown below (*blue-adult*; *magenta-aged*). Bottom: Histograms show normalized unit firing probability measured in 1 ms delay bins relative to the negative peak of CA3 ripples (dashed lines). Note that several peaks occur at approximately 5 ms intervals, corresponding to the period of the ripple cycle. **C**. Top: CA3 ripple waveform averages. Bottom: Mean normalized unit firing probabilities. Note the reduced firing probability during the earlier ripple cycles in the aged. Inset: Scatter plot of the delay bins containing the peak firing probabilities for adult neurons (*blue*) and aged neurons (*magenta*). The median delay was significantly larger in aged slices compared to adult slices. For clarity, dots were offset along the categorical axis. *p<0.05.

Network timing alterations appeared to be restricted to the stratum pyramidale/stratum oriens layers of aged CA1, which might reflect changes in the spiking patterns of local neurons. To test this hypothesis, we obtained juxtacellular recordings from neurons near the stratum pyramidale/stratum oriens border in CA1 and measured unit timing in relation to ripple oscillations recorded simultaneously from CA3 (aged = 14 neurons from 5 mice, adult = 15 neurons from 5 mice; Fig. 4A). Most neurons fired one or two spikes during each sharp wave ripple (aged = 10 of 14 neurons, adult = 11 of 15 neurons), with four neurons from each group consistently firing bursts of three to seven spikes. Units occurred predominately on the ascending phase of individual ripple cycles (circular mean±SD: aged = 1.98±0.28 radians, p<0.001; adult = 2.26±0.17 radians, p<0.001, Rayleigh test), and these phase relationships did not differ between age groups (t_27_ = 0.82, p = 0.42, Watson-Williams test). Additionally the “depth of modulation” (i.e. resultant vector length) was comparable between age groups (aged = 0.27±0.05, adult = 0.34±0.04, t_27_ = 1.06, p = 0.30, t-test). Whereas the spike timing relationship to the ripple cycle was fairly homogenous, the peak firing probabilities of individual CA1 neurons occurred at different times relative to the peak negativity of the CA3 ripple oscillation (Fig. 4B). A Mann-Whitney U test indicated that the median peak firing delay was significantly larger in aged slices vs. adult slices (aged = 8 ms, adult = 3 ms, U = 48, p = 0.012; Fig. 4C *inset*). On average, aged CA1 neurons exhibited reduced spike probability during the early cycles of CA3 ripple oscillations (Fig. 4C, compare 95% confidence intervals approximately -3 ms relative to the ripple's peak negativity). Thus, the increased time delay associated with network activity entrained in aged CA1 was also evident in the spiking activity entrained in this subregion.

## Discussion

This study used field potential recordings from hippocampal slices to examine the effects of aging on network timing in the CA3–CA1 circuit. The magnitude of sharp wave ripple activity recorded near the stratum pyramidale/stratum oriens border of CA1 was smaller in aged slices and its relative timing was delayed when compared to adult slices. Carbachol-induced gamma power was reduced in aged CA3, as was CA3–CA1 coherence. Furthermore, the phase difference between CA3 and CA1 gamma was increased in the aged. Therefore, aging appears to alter the relative timing of network activity entrained in CA1. To identify a potential mechanism for this finding we examined Schaffer collateral responses elicited by electrical stimulation delivered near the CA3–CA1 border in stratum radiatum. Schaffer collateral evoked antidromic spikes recorded in CA3 stratum pyramidale were smaller in aged slices, as were the slopes of orthodromic field EPSPs recorded in CA1 stratum radiatum. However, aging did not alter the amplitude or timing of the spontaneous sharp wave field EPSP recorded in CA1 stratum radiatum. Instead, it appears that the network timing alteration was restricted to the stratum pyramidale/stratum oriens border of CA1. In this regard, juxtacellular unit recordings from aged neurons in this region revealed reduced spike probabilities during the early cycles of ripple oscillations recorded upstream in CA3. Combined, these data provide *in vitro* evidence of an age-related impairment in coordinated activity in CA3–CA1 circuit. We discuss these findings below in the context of previously reported data on age-related changes hippocampal physiology.

Some of our results are consistent with earlier reports of age-related alterations in hippocampal network activity. For example, CA1 ripple energy was reduced in slices from aged mice examined in another study [20]. However, in that study only ripple energy was reduced, whereas we found reductions in the intensity of both ripples and sharp waves in CA1. Also, in our study CA3 but not CA1 gamma power was reduced in the aged slices, whereas in an earlier report aged slices exhibited reduced power in both subregions [22]. Some of these discrepancies might be explained by differences in the genetic backgrounds of the mice used in these studies 31] and could also reflect differences in the methods used to quantify power. However, all the reports support the findings of age-related impairments in hippocampal network function.

The results of our study do not identify a clear mechanism for the network alterations observed in the aged slices, and could reflect a number of changes in hippocampal physiology associated with advanced age. For instance, the strength of synaptic excitation is reportedly reduced in the CA1 subregion of aged rats [24], [25], [26], which might weaken CA3–CA1 network synchrony as hypothesized. However, while Schaffer collateral evoked field EPSP slopes were reduced, we found no difference between age groups in the amplitude of spontaneous field EPSPs, suggesting that the strength of synaptic excitation associated with sharp waves was not altered in the aged slices. Slower conduction velocity could theoretically account for the observed timing alterations. Although we did not measure Schaffer collateral conduction velocity directly, the latency of the antidromic spike recorded in CA3 was similar in both age groups. However, the reduction in spike amplitude suggests possible reductions in the number or excitability of Schaffer collateral afferents. Unfortunately, fiber volleys were not present in the field EPSP responses recorded in our study, but this measure was not altered in another study [32]. Overall, while impaired Schaffer collateral transmission might contribute to some of our findings, we think this possibility is unlikely given that the timing and amplitude of sharp wave associated field EPSPs were similar in both age groups.

Reduced neuronal excitability might account for some of our findings. Aging increases L-type calcium channel activity [33] and alters other aspects of calcium homeostasis in the hippocampus [34]. Calcium dysregulation is believed to result in increased conductance of the calcium dependent after-hyperpolarizing potassium current (I_AHP_) and decreased neuronal excitability [35], [36], [37]. Gamma power deficits have been associated with increases in the amplitude of the slow AHP recorded from pyramidal cells and interneurons in CA3 [21], [23]. Additionally, a recent study described a∼3 mV depolarizing shift in the action potential threshold recorded from aged CA1 pyramidal cells, which was accompanied by a similar shift in the activation of voltage gated sodium channels [38]. In this regard, the decreased spike probability we observed during the early ripple cycles might reflect failure of a subset of aged neurons to reach spike threshold. This hypothetical subset of neurons might require more time to integrate Schaffer collateral EPSPs in order to reach threshold, and the time delays manifested in the network activity could reflect the delayed synaptic responses generated by these late-spiking neurons.

A number of anatomical reports have also described age-related changes in various interneuronal markers in the hippocampus, which could help explain some of our findings given the important role synaptic inhibition plays in generating hippocampal network patterns. For example, there is a widespread reduction in glutamic acid decarboxylase (GAD) expression in the aged hippocampus, although the number of interneurons [39] and the number of GAD-immunoreactive boutons [40] are not reduced. There also appears to be a preferential reduction in the number of somatostatin-immunopositive neurons in aged CA1, whereas parvalbumin-immunopositive neurons are relatively preserved [27], [28], [41]. Although the somatostatin neurons were presumed to represent oriens lacunosum-moleculare (O-LM) cells, which provide distal dendritic inhibition coaligned with entorhinal input, there is another subclass of somatostatin-immunopositive neuron, the so called “oriens-bistratified” cells, which also have cell bodies and horizontal dendritic arbors in stratum oriens like O-LM cells, but have axonal projections coaligned with Schaffer collateral inputs in stratum radiatum and stratum oriens [42], [43]. Bistratified cells fire action potential phase locked to gamma oscillations [44] and ripple oscillations [45], and can be monosynaptically activated by CA3 Schaffer collaterals [46], [47], suggesting they could play an important role in entraining CA3 network patterns in CA1. It is also interesting to note that bistratified cells begin firing early during the ripple oscillation [45]. Although it is unclear whether bistratified cells are also lost with age, it is possible that the age-related decrease in unit firing probability we observed early in the ripple oscillation reflects a preferential loss of this subpopulation of interneuron. However, this is very speculative since we did not determine the identity of the units recorded in the present study.

A potentially important limitation of our study is the use of only two electrodes to assess network interactions between CA3 and CA1. We chose CA3c and mid-distal CA1 based on preliminary studies in which we observed that sharp waves and gamma oscillations were largest in these subregions. It is unclear whether aging also disrupts network timing between other portions of the CA3–CA1 circuit; multi-electrode arrays could be used to answer this question. Unfortunately, we could not differentiate pyramidal cells from interneurons by their waveform characteristics [48] because of the low sampling resolution (5 kHz), which resulted in units with truncated waveforms. A higher sampling resolution combined with juxtacellular labeling might have provided enough information for us to determine whether the age-related alteration in ripple-modulated spiking in CA1 involved a specific subpopulation of neuron (i.e. pyramidal cells vs. interneurons). As with any *in vitro* experiment, the relevance of these findings to the intact brain is uncertain. It will be important to determine whether aging disrupts CA3–CA1 network timing *in vivo*, and if so, whether the magnitude of the deficit is associated with hippocampus-dependent memory impairment.

In summary, CA3-entrained network and single cell activity recorded near the cell body layer of CA1 was temporally delayed in slices from aged mice. Successful reactivation and retrieval of information during sharp wave ripples and gamma oscillations, respectively, may require that cell assemblies distributed between CA3 and CA1 be coactive within a certain window of time in order to modify synaptic strength and/or generate action potentials to propagate information through the hippocampal formation. It is possible that a contributing factor to age-related memory impairment involving the hippocampus is a failure to bind information partitioned between CA3 and CA1.

## Supporting Information

Figure S1 **CA3–CA1 sharp wave timing estimated by cross-correlation.** Plots show mean cross-correlations between CA3 and CA1 along with the estimated 95% confidence intervals (*adult-blue; aged-magenta*). The time difference estimated from the peaks of the cross correlation was significantly larger in aged slices compared to adult slices. Values represent mean ± SEM. **p<0.01.
(TIFF)

Figure S2 **CA3–CA1 gamma timing estimated by cross-correlation.** Plots show mean cross-correlations between CA3 and CA1 along with the estimated 95% confidence intervals (*adult-blue; aged-magenta*). The time difference estimated from the peaks of the cross correlation was significantly larger in aged slices compared to adult slices. Values represent mean ± SEM. **p<0.01.
(TIFF)
