# Supplementary figures and images for: Altered Network Timing in the CA3-CA1 Circuit of Hippocampal Slices from Aged Mice
Source: PLoS One. 2013 Apr 8;8(4):e61364. doi: 10.1371/journal.pone.0061364 (PMC3620228; doi:10.1371/journal.pone.0061364)

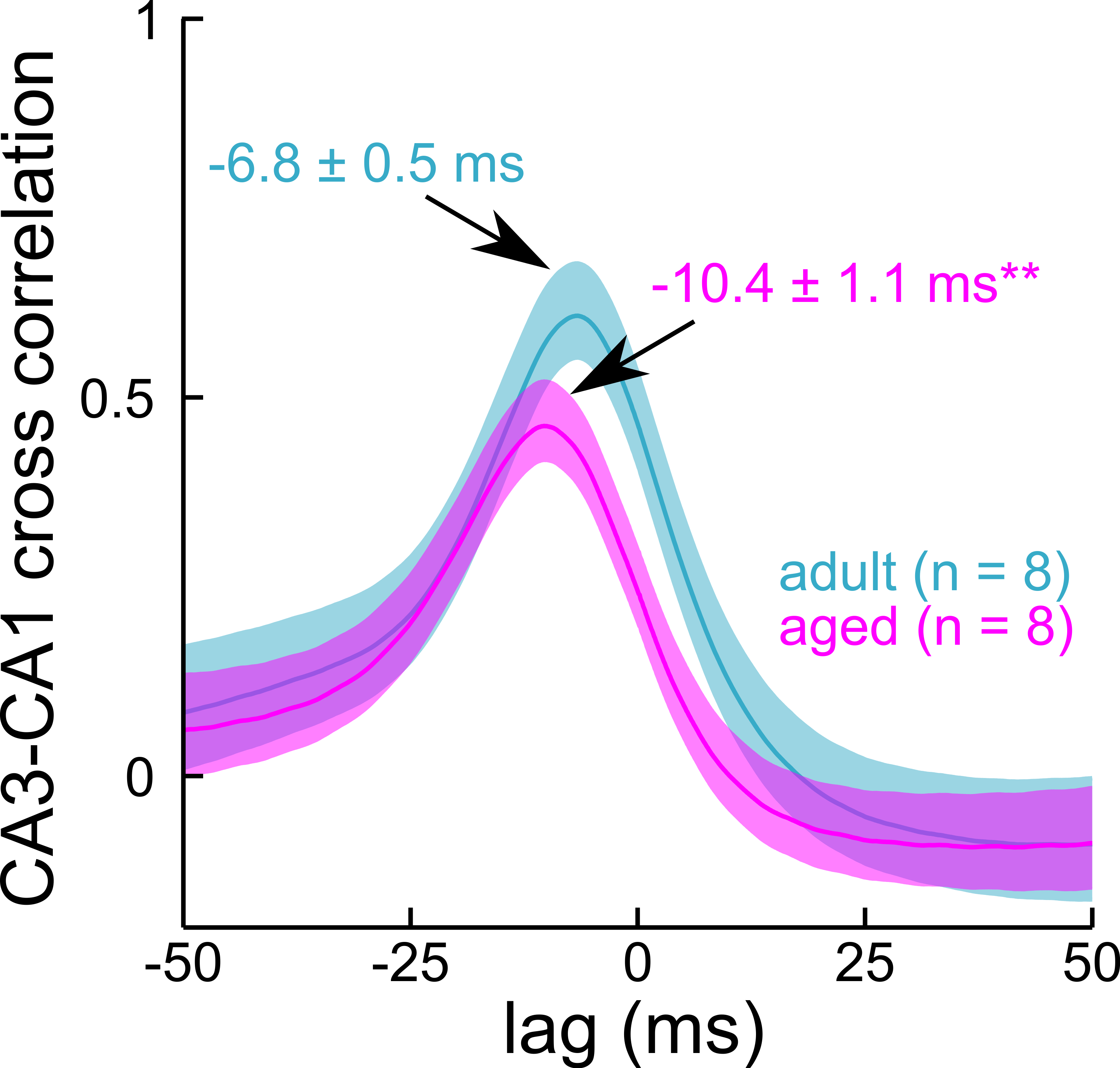

Supplement: Figure S1 — CA3–CA1 sharp wave timing estimated by cross-correlation. Plots show mean cross-correlations between CA3 and CA1 along with the estimated 95% confidence intervals (adult-blue; aged-magenta). The time difference estimated from the peaks of the cross correlation was significantly larger in aged slices compared to adult slices. Values represent mean ± SEM. **p<0.01. (TIFF) [file pone.0061364.s001.tiff]

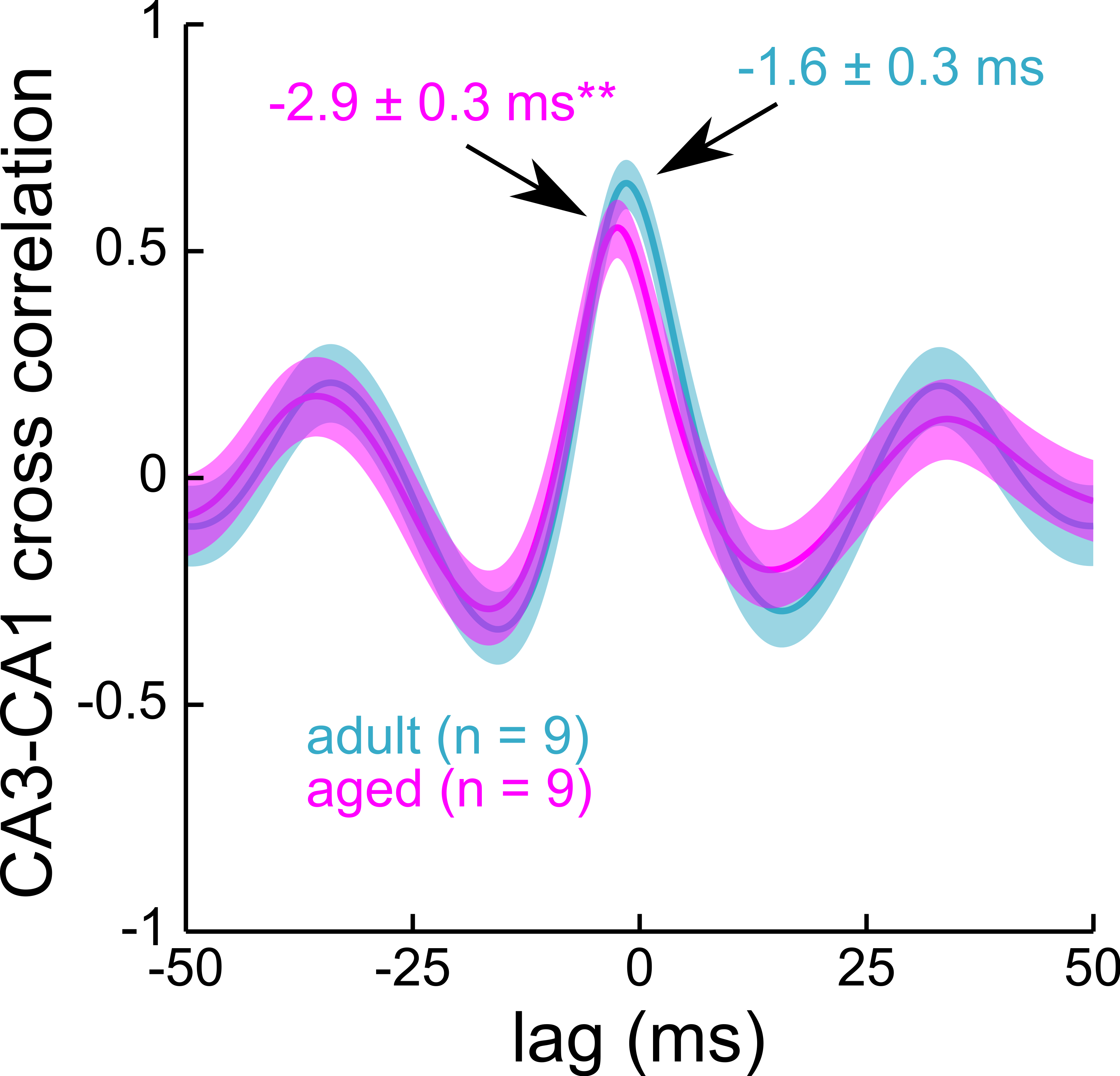

Supplement: Figure S2 — CA3–CA1 gamma timing estimated by cross-correlation. Plots show mean cross-correlations between CA3 and CA1 along with the estimated 95% confidence intervals (adult-blue; aged-magenta). The time difference estimated from the peaks of the cross correlation was significantly larger in aged slices compared to adult slices. Values represent mean ± SEM. **p<0.01. (TIFF) [file pone.0061364.s002.tiff]
